# Surgical Management of Freiberg Disease by Dorsal Closing Wedge Osteotomy

**DOI:** 10.5704/MOJ.2011.025

**Published:** 2020-11

**Authors:** SA Dhar, NA Mir, TA Dar

**Affiliations:** Department of Orthopaedics, Sher -i - Kashmir Institute of Medical Sciences (SKIMS) Medical College Bemina, Srinagar, India

**Keywords:** Freiburg infraction, osteotomy

## Abstract

**Introduction:**

The purpose of the study was to assess the efficacy of the dorsal closing wedge osteotomy for the treatment of Freiburg’s infraction.

**Material and Methods:**

Twenty patients with Freiburg’s infraction were admitted at our hospital over a period of six years. Patients with a normal plantar contour of the metatarsal head were included. All patients underwent a dorsal closing wedge osteotomy of the metatarsal.

**Results:**

The mean Leeds Movement Performance Index (LMPI) score was 84 (range 70-86). The mean metatarsal shortening was 2mm. the passive flexion restriction was 16° and extension restriction was 10°. Also, a strong negative correlation was found between Smillie classification and American Orthopaedic Foot and Ankle Score (AOFAS) final score (r’s = −0.85, P < .001).

**Conclusion:**

The dorsal closing wedge osteotomy is an efficient and reproducible method for the management of Freiburg’s infraction.

## Introduction

**Fig. 1:**
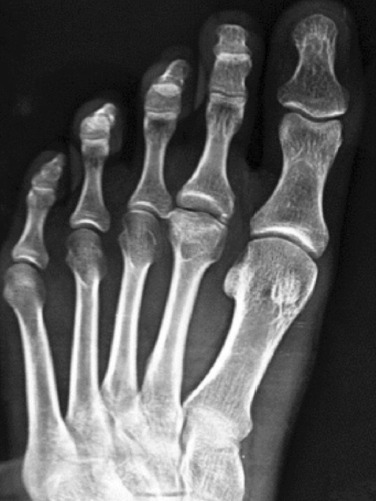
Freiburg’s infraction of the second metatarsal.

The Freiberg’s infraction is the painful collapse of the articular surface of the metatarsal head (Fig. 1)^1^. The disease affects females predominantly^2^. The second metatarsal is most commonly affected^3^. Various theories have been proposed in the etiology of this disease. These include injury to the vascular supply of the metatarsal head and trauma which may be a single event or a repetitive phenomenon^4,5^. The patients tend to report pain on walking or a feeling of a small hard object under the sole of the foot. Freiberg’s infraction can be treated conservatively if detected early, but late presentation may have to be managed by surgery^6^. Several conservative methods have been used in the management of Freiberg’s infraction. These are activity modification, insoles, metatarsal pads, casting and controlled ankle motion boots^7^. Surgical interventions include debridement, bone grafting, interpositional arthroplasty, core decompression or joint replacement^8^. One of the methods used for the management of Freiberg’s infraction is the dorsiflexion osteotomy of the metatarsal neck which rotates the cartilage facing plantarwards into the joint thus improving the arthrosis.

This study reports the results of this procedure done on 20 patients over a five years period in our department.

## Materials and Methods

**Fig. 2:**
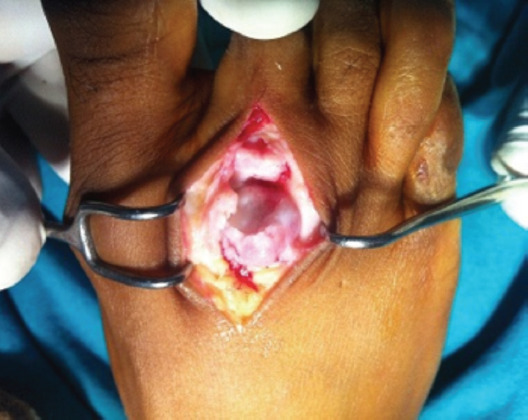
The damaged distal part of the head of the metatarsal.

**Fig. 3:**
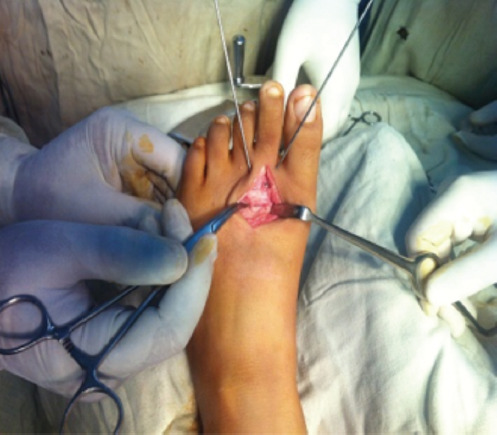
The closed osteotomy.

Twenty patients presenting to the outpatient department (OPD) of the SKIMS Medical College Bemina were assessed to have stage 4 and 5 Smillie grade infraction with good plantar contour and cartilage of the metatarsal head. Under anaesthesia, an incision (3.5cm) was made dorsomedial to the extensor digitorum longus tendon from the metatarsal neck to the base of the phalanx. Superficial and deep fascia were dissected carefully and the extensor tendon was retracted laterally. The capsule was opened dorsally in a longitudinal fashion followed by a cheilectomy and synovectomy (Fig. 2). A wedge-shaped osteotomy was done with the distal cut being made 2-3mm proximal to the unhealthy part and the distal fragment was rotated dorsally so that the volar surface is brought distally (Fig. 3). We used a K-wire as a joystick to control the distal fragment into a proper position as it is difficult to control the distal fragment otherwise. The angle of the osteotomy was 15° but was increased if the rotation required was more. The osteotomy was held by one or two k-wires. The k-wire/wires were removed at six weeks.

## Results

**Fig. 4:**
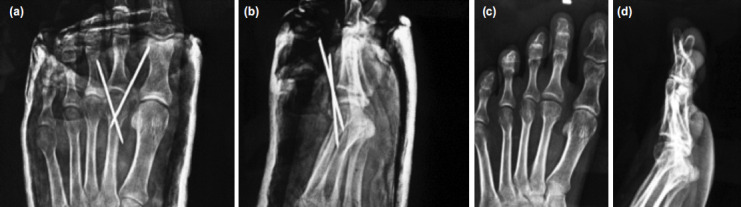
Post-operative radiographs; (a) anteroposterior, (b) lateral. Radiograph at final follow-up (c) anteroposterior and (d) lateral views.

**Fig. 5:**
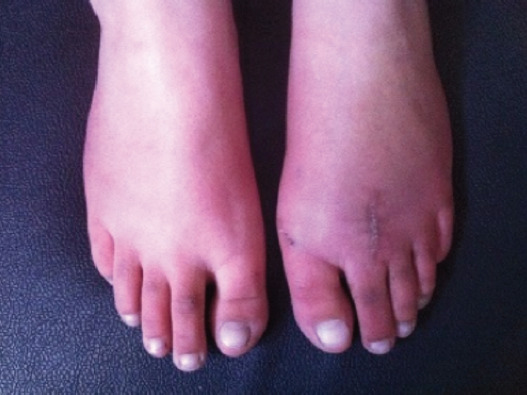
Clinical picture with mild shortening.

The average follow-up of the patients was 12 to 60 months. All cases united at six weeks but protected weight bearing was continued for 12 weeks (Fig. 4 and 5). The average metatarsal shortening was 1.75mm (range of 1-4mm) due to the osteotomy. The results were graded as per the LMPI scale which allots 40 points to pain, 45 points to function and 15 points to function (maximum 100 points)^9^. The mean LMPI score was 84 (range 70-86). The mean metatarsal shortening was 2mm, the passive flexion restriction was 16° and extension restriction was 10° (Table I).

**Table I:** The pre-operative and post-operative assessment

S No	Age	Side	Metatarsal affected	Stage (Smillie)	Symptoms (Pre-operation)	Shortening (mm)	Range of motion Pre / Post	LMPI score Pre / Post
1.	19	R	2	5	pain/ hard object	1	40 / 60	52 / 70
2.	23	L	2	4	pain	4	20 / 40	57 / 86
3.	18	L	2	4	pain	2	25 / 40	62 / 80
4.	27	L	2	4	pain	2	25 / 35	57 / 80
5.	34	L	3	4	pain	2	25 / 40	72 / 82
6.	30	R	2	4	pain	1	30 / 50	75 / 85
7.	17	R	2	4	pain	1	35 / 50	72 / 86
8.	17	L	2	5	pain/ hard object	1	30 / 40	57 / 80
9.	17	R	2	5	pain	1	25 / 40	62 / 86
10.	23	L	3	5	pain	2	25 / 40	75 / 86
11.	28	L	2	4	pain	2	30 / 60	27 / 84
12.	33	R	3	4	pain	1	25 / 40	57 / 84
13.	26	R	2	4	pain	2	30 / 55	62 / 84
14.	32	R	2	5	pain	4	20 / 40	57 / 86
15.	19	R	2	5	pain	3	35 / 55	62 / 85
16.	20	L	3	5	pain	1	25 / 40	27 / 85
17.	21	R	2	5	pain	1	30 / 45	62 / 85
18.	19	R	2	4	pain	1	30 / 60	57 / 86
19.	16	L	2	4	pain	1	25 / 40	57 / 86
20.	16	L	2	5	pain/ hard object	2	30 / 55	62 / 86

Also, a strong negative correlation was found between Smillie classification and AOFAS final score (r’s = −0.85, P < .001) (Table II).

**Table II:** Smillie staged the osteochondrosis into five stages^13^

Smillie staging
Fissure type fractures in an ischaemic epiphysis. Altered articular contour of the dorsal and central part of the head Altered articular contour with widening and subchondral cystic changes. Presence of loose bodies. Complete metatarsal head flattening and a deforming arthrosis.

## Discussion

Freiberg’s infraction is the second most common osteochondrosis of the foot. The disease has also been referred to as ‘egg shell fracture’, ‘metatarsal epiphysitis’, ‘osteochondritis deformans metatarsojuvenilis’ and ‘malakopathie’^10,11^. This probably points to the persistent debate over the etiology of the disease.

Freiberg’s infraction is always situated at the front, dorsal part of the metatarsal head, and is considered to be a dorsal trabecular stress injury of the second or the third metatarsal head. Excessive pressure on the metatarsal head during weight bearing could cause repetitive microfracture, loss of blood supply to the subchondral bone, collapse of the cancellous bone, and cartilage deformation^12^.

Smillie staged the osteochondrosis into five stages^13^. First, fissure type fractures in an ischaemic epiphysis. Second, altered articular contour of the dorsal and central part of the head. Third, altered articular contour with widening and subchondral cystic changes. Fourth, presence of loose bodies and fifth, complete metatarsal head flattening and a deforming arthrosis.

The recommended method of treatment of stages 4 and 5 is surgery. The goal is to restore joint congruence and motion^14^.

Some surgical procedures are fraught with complications. Resection arthroplasty can cause transfer metatarsalgia. Should conservative treatment fail, a wide variety of surgical procedures exist; however, the optimal procedure is unknown. A review published in 2015 reported that only 257 joint sparing procedures have been reported in literature pointing to a relative paucity of literature in this regard^15^.

**Fig. 6:**
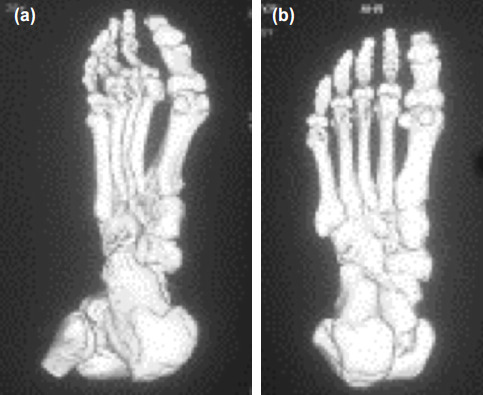
The shaded surface display on CT scan showing a good plantar contour of the second metatarsal; (a) oblique view and (b) inferior view.

The extent of necrosis is the main determining factor in the outcome of osteonecrosis. There is no method for measuring the extent of osteonecrosis of the metatarsal head in Freiberg’s infraction. The use of CT scanning helps in assessment of the extent and allows the surgeon to plan a procedure in a better manner (Fig. 6)^16^.

**Fig. 7:**
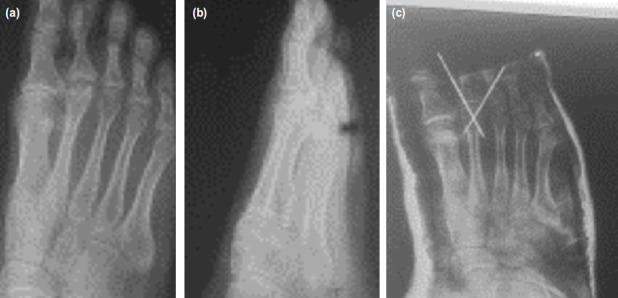
The radiographs show osteotomy where no debridement was done; (a) anteroposterior view, (b) lateral view and (c) postoperative view.

Dorsal closing wedge osteotomy was reported by Gauthier *et al*^3^. Some series have been reported since with the partial modification of replacing the cerclage wire used originally by a k-wire^17,18,19^. The dorsiflexion osteotomy realigns the intact plantar metatarsal cartilage and hence provides a more physiological joint congruence and motion. The procedure also allows decompression with minimal shortening. Gauthier and Elbaz removed unhealthy tissue during dorsal wedge closing osteotomy, but it has been found that the remaining intact portion of the metatarsal head was too small for internal fixation to be performed. Thus, the osteotomy site is moved more proximally to allow a better purchase and fixation. We did not debride the lesion in any of our cases (Fig. 7).

This technique has good results, and its complications are in most cases related to the use of osteosyntesis material. With the development of percutaneous surgery, this wedge osteotomy could be performed through a minimal incision, reducing morbidity. The procedure is also reproducible and the use of a k-wire allows removal of hardware on an outpatient department basis.

## Conclusion

The dorsal closing wedge osteotomy is an efficient and reproducible method for the management of Freiburg’s infraction.
